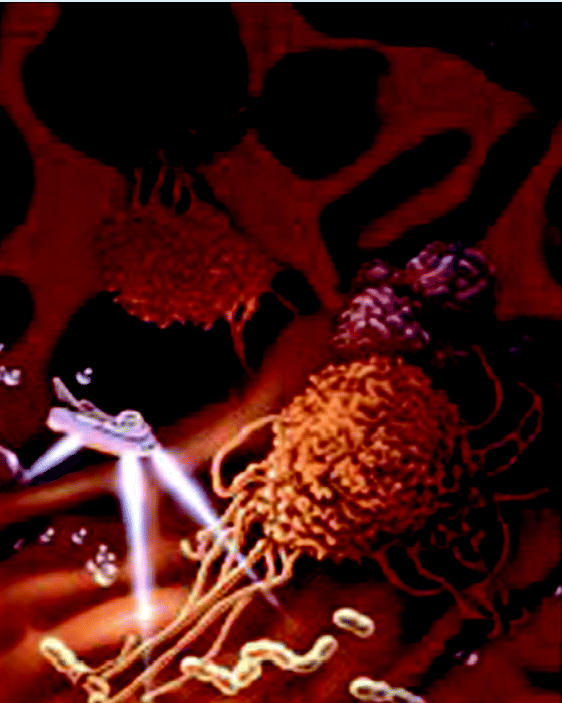# Nanotechnology in the Environmental Health Sciences

**Published:** 2005-05

Among the newest buzzwords in biomedical science is nanotechnology: small is big! The vision for nanotechnology has existed for many years—remember Isaac Asimov’s *Fantastic Voyage*—but the ability to manipulate individual atoms to engineer devices at the nanoscale is new. A number of benefits arise at the nanoscale, from the practical (reagent utilization and the ability to multiplex) to the emergence of new properties (optical and electrical). The intent here is to briefly outline some ways nanotechnology will impact the environmental health sciences.

## Sensor Technologies

The benefits of nanotechnology make it ideal for sensor development, for environmental and biological monitoring as well as for linking exposure, disease, and susceptibility. Investigators are developing arrays for toxicants based on technologies such as ion channels or fluorescence-emitting nanoprobes. Similarly, nanomaterials are being used to investigate the mechanisms of disease etiology *in vitro* and *in vivo*.

## Remediation Technologies

Nanomaterials offer two distinct advantages to remediation technologies: large surface-area-to-volume ratio and high chemical reactivity. This pays dividends for both catalysis (for example, with halogenated organics) and sequestration (for example, with radionuclides). One key issue needing to be resolved is how nanomaterials behave in the environment as they are used for site remediation.

## Nanoparticle Toxicity

There are indications that exposure to certain nanomaterials may lead to adverse biological effects that appear to depend on the material’s chemical and physical properties. Nanoparticles will interact with biological systems; issues such as particle absorption and the contributions of surface geometry, chemistry, cellular uptake, and localization need to be examined to inform toxicity assessments for nanomaterials.

## Long-Range Vision

Our ultimate “blue sky” goal is similar to the vision Asimov presented 40 years ago—to develop brilliant biocompatible nanoparticles to be used *in vivo* to detect exposure to a potential toxicant, to identify biological responses to that exposure and categorize them as compensatory or pathological, and to intervene to halt or reverse the development of disease.

## Contact

**David Balshaw, Phd** | balshaw@niehs.nih.gov

**Sally Tinkle. PhD** | stinkle@niehs.nih.gov

## Figures and Tables

**Figure:**